# A Tolerogenic Role of Cathepsin G in a Primate Model of Multiple Sclerosis: Abrogation by Epstein–Barr Virus Infection

**DOI:** 10.1007/s00005-020-00587-1

**Published:** 2020-06-16

**Authors:** Bert A. ‘t Hart

**Affiliations:** 1 Department of Biomedical Sciences of Cells and Systems, University of Groningen, University Medical Center, Groningen, The Netherlands; 2 Department of Anatomy and Neurosciences, VU Medical Center, Amsterdam, The Netherlands

**Keywords:** EAE, Autoimmune, Tolerance, B cells, Antigen presentation

## Abstract

Using a non-human primate model of the autoimmune neuroinflammatory disease multiple sclerosis (MS), we have unraveled the role of B cells in the making and breaking of immune tolerance against central nervous system myelin. It is discussed here that B cells prevent the activation of strongly pathogenic T cells present in the naïve repertoire, which are directed against the immunodominant myelin antigen MOG (myelin oligodendrocyte glycoprotein). Prevention occurs via destructive processing of a critical epitope (MOG34-56) through the lysosomal serine protease cathepsin G. This effective tolerance mechanism is abrogated when the B cells are infected with Epstein–Barr virus, a ubiquitous γ1-herpesvirus that entails the strongest non-genetic risk factor for MS.

## Introduction

During fetal development, the immune system is instructed to react against foreign agents (non-self antigens) while ignoring components from the host’s body (self antigens). The thymus has a central role in this process, as T cells competent to react against self are eliminated from the repertoire via negative selection, while T cells recognizing non-self antigens presented by the host’s major histocompatibility complex (MHC) molecules are allowed to enter the repertoire via positive selection (Nossal 1991). Nevertheless, studies in laboratory animals (mice, rats, primates) revealed that T cells capable of inducing autoimmune-driven neuroinflammatory disease are present in the healthy immune repertoire, suggesting that these autoreactive specificities have escaped thymic (negative) selection (Ben-Nun et al. 1981; Meinl et al. 1997; Schluesener and Wekerle 1985; Villoslada et al. 2001). Using the well-validated experimental autoimmune encephalomyelitis (EAE) model in common marmosets (*Callithrix jacchus*), a small bodied Neotropical primate, we have explored how pathogenic T cells specific for the pathogenically relevant myelin antigen myelin oligodendrocyte glycoprotein (MOG) (Jagessar et al. 2008) are maintained inactive in healthy animals and how they are activated under conditions relevant to multiple sclerosis (MS), the human disease on which the EAE model has been projected. This short review gives a concise overview of these studies.

## The EAE Model in Common Marmosets

EAE in common marmoset monkeys (*Callithrix jacchus*) is a validated animal model of the human autoimmune neuroinflammatory disease MS (‘t Hart et al. 2015). The model has a high face validity for MS as it replicates essential clinical and pathological aspects of the human disease (‘t Hart et al. 1998). Moreover, evidences from immunotherapy and mechanistic studies performed over the past two decades reveal a high construct validity, indicating that pathogenic mechanisms operating in the model are representative for the human disease (Kap et al. 2016). These features underscore the translational relevance of the model for research into pathogenic mechanisms as well as therapy development.

Recent work shows that the model is potentially useful for studies on the biological underpinning of factors that increase the risk of developing MS, such as infection with Epstein–Barr virus (EBV) (‘t Hart et al. 2013). EBV is a γ1-herpesvirus that infects human B lymphocytes via binding to complement C3d receptor (CD21) (Fingeroth et al. 1984). Importantly, the marmoset carries a natural infection with an EBV-related γ1-herpesvirus called CalHV3 that has comparable effects on the B cells (Cho et al. 2001).

After the discovery that B cell depletion via a monoclonal antibody (mAb) directed against the B lineage specific marker CD20 has a profound clinical effect in MS, the B cell has gained profound interest as a relevant target of therapy (Hauser et al. 2008). Newly recognized pathogenic functions of B cells beyond their traditional role, being production of autoantibodies that opsonize myelin, are cytokine production, the organization of ectopic lymphoid structions within the central nervous system and antigen presentation to T cells (von Budingen et al. 2015). This short review will discuss data on the latter role of B cells obtained in the marmoset EAE model.

## B Cells as Crucial Antigen-Presenting Cells in MS and Its Animal Model EAE

Marmosets immunized with myelin isolated from the brain of an MS patient, which was obtained via the Netherland’s brain bank (Amsterdam, Netherlands), developed an inflammatory demyelinating autoimmune disease that shows remarkable clinical and pathological similarities with MS (‘t Hart et al. 1998; Absinta et al. 2016). Our studies in mice and marmosets revealed that among the multitude of candidate myelin autoantigens, the quantitatively minor myelin component MOG has a central immunopathogenic role (Jagessar et al. 2008; Smith et al. 2005). In a marmoset EAE model elicited with recombinant human (rh) MOG, two peptides located in the Ig-like extracellular domain were found to contain immunodominant T cell epitopes, namely MOG14-36 (residues 24–36 identified as epitope for MHC class II/Caja-DRB*W1201-restricted CD4^+^ T cells (Brok et al. 2000)) and MOG34-56 (residues 40–48 identified as epitope for MHC class Ib/Caja-E-restricted CD8^+^CD56^+^ T cells (Jagessar et al. 2012b)). The two peptides elicited distinct pathogenic mechanisms, which to some extent represent the relapsing–remitting and progressive phases of MS, respectively (‘t Hart et al. 2011).

Our studies revealed a central pathogenic role of B cells in marmoset EAE as late-stage depletion (from post-immunization day 21 onward) with a clonal variant of the clinically tested anti-CD20 mAb ofatumumab-suppressed clinical EAE development in marmosets sensitized against the full-length rhMOG protein (Kap et al. 2010, 2011) or the immunodominant peptide MOG34-56 (Jagessar et al. 2012a). A model induced with the MOG34-56 peptide is of particular interest, as the expression of MS-like pathology and clinical signs in this model is driven by the interaction of B cells with cytotoxic CD8^+^ CD56^+^ effector memory T cells having IL-17A as cytokine signature (‘t Hart et al. 2017). Notably, MS pathology in the MOG34-56-induced model developed in the absence of antibodies capable of binding myelin, as these were not detectable in the circulation (Jagessar et al. 2015).

The promising clinical effect of anti-CD20 mAbs sparked tests of other B cell depletion strategies. It was found in the rhMOG-induced marmoset EAE model that treatment with antibodies capturing cytokines that mediate survival and differentiation of B cells, i.e., BLyS and APRIL, did lead to B cell depletion from the peripheral blood, but exerted only a marginal effect on the expression of EAE symptoms or pathology (Jagessaret al. 2012a). Atacicept is a chimeric construct consisting of the Fc tail of human IgG and the joint receptor of BLyS and APRIL. This study in the EAE model essentially replicated the failure of atacicept in relapsing MS clinical trials (Kappos et al. 2014). The reason for this discrepant clinical effect was examined in the marmoset EAE model, revealing that treatment with anti-BLyS and anti-APRIL antibodies failed to deplete CalHV3 (-infected B cells) from the lymphoid system, while treatment with anti-CD20 mAb depleted also this subset (Jagessar et al. 2013). These findings led us to hypothesize that a critical pathogenic effect of B cells is mediated by a small-sized subset of CalHV3-infected B cells (< 0.05% of all B cells) (Khan et al. 1996), which present a key pathogenic peptide (MOG34-56) to autoaggressive T cells. This finding might give a plausible mechanistic explanation for the close association of EBV infection and MS risk (‘t Hart et al. 2017).

## Cathepsin G as a Leading Protease in the Processing of MOG in (EBV-Infected) B Cells

We observed that immunization with rhMOG in the mineral oil IFA induced MS-like pathology in three non-human species, indicating that autoreactive T cells present in their naïve repertoire can be activated without the normally requisite co-stimulation by danger signals (Haanstra et al. 2013). Immune profiling of this atypical EAE model in marmosets revealed that T cell and antibody reactivity against several MOG peptides, i.e., MOG14-36, MOG24-46 and MOG54-76, could be detected while reactivity of blood mononuclear cells and serum with the MOG34-56 peptide was conspicuously absent (Jagessar et al. 2015). However, the model induced with MOG34-56/IFA demonstrates that T and B cells reactive with MOG34-56 peptide are present in the marmoset’s repertoire (Jagessar et al. 2010). Hence, we hypothesized that the peptide might be destroyed during processing in antigen-presenting cell (APC), being (CalHV3-infected) CD20^+^ B cells, probably as an effective strategy to avoid activation of autoreactive T cells that have escaped negative selection in the thymus (Manoury et al. 2002).

Experiments testing the degradation of rhMOG and MOG34-56 in lysates of non-human primate CD20^+^ spleen cells in the presence of protease inhibitors revealed that the degradation of both proteins is inhibited by addition of the cathepsin G antagonist CMK (Jagessar et al. 2016), indicating that cathepsin G may lead to autoantigen degradation, as was observed earlier by Burster et al. (2004) for another myelin antigen (MBP). Cathepsin G is a serine protease that has gained multi-specificity during mammalian evolution due to mutation of specificity-determining residues at positions 189 and 226 (Raymond et al. 2010). While cathepsin G of mice (Ser^189^/Ala^226^) has narrow chymotrypsin-like specificity for some aromatic amino acids (Phe and Tyr), cathepsin G of humans (Ala^189^/Glu^226^) has a broader specificity range, including also Arg, Leu, His, Trp, Lys, and Met. The paper claims that cathepsin G of marmosets (Thr^189^/Ala^226^) has similar narrow chymotryptic activity as the mouse protease. Nevertheless, we observed that the degradation of the MOG34-56 peptide was abrogated when the Arg residues at positions 41 and 46 (underlined) were replaced by citrulline, despite the presence of at least three additional potential cleavage sites (bolded): GMEVGW**YR**PP**F**SRVVHL**Y**RNGKD (Jagessar et al. 2016).

The highlighted (yellow) sequence (^40^YRPPFSRVV^48^) represents the epitope of MHC-Ib/Caja-E-restricted CD8^+^ CD56^+^ T cells that accelerate EAE progression in the rhMOG/CFA model and forms the core pathogenic activity in the MOG34-56/IFA model (Jagessar et al. 2010, 2012b; Kap et al. 2008). The epitope seems to be a hot-spot of proteolytic activity as, besides the above-discussed cleavage by cathepsin G, it may also be a target of TSSP, a thymus-specific serine protease involved in the processing of antigen expressed by thymic epithelial cells. TSSP is a proline-specific dipeptidase that was found to abort negative thymic selection of MOG34-56-specific encephalitogenic T cells in C57BL/6 mice by destructive processing of the MOG40-48 epitope in thymic epithelial cells (Serre et al. 2017). It is tempting to speculate that the destructive processing of the same epitope by cathepsin G in B cells may function as a peripheral backup system for preventing activation of encephalitogenic T cells that have escaped thymic selection.

## The Effect of EBV Infection on APC Function of B Cells

The central question arising from the in vivo experiments in the marmoset EAE model has been: why does infection of B cells with EBV enhance their APC function? A critical finding has been that the MOG40-48 epitope is cross-presented via Caja-E to the CD8^+^ CD56^+^ CTL (Jagessar et al. 2012b). It has been documented that B cells are poorly capable of antigen cross-presentation, unless they are infected with a γ1-herpesvirus (Jagessar et al. 2012b) or stimulated via TLR9 with CpG (Jiang et al. 2011).

Among the multiple changes that EBV infection causes in B cells, two processes seem to be of particular importance, namely the induction of peptidyl-arginine deïminase (PAD) activity and the activation of autophagy (Jagessar et al. 2016; Morandi et al. 2017). The importance of increased PAD activity is that peptide citrullination, altering antigen processing (Jagessar et al. 2016), can take place within EBV-infected B cells (Ireland and Unanue 2011). The induction of autophagy may be of particular interest, as epitope loading of MHC-E molecules seems not to occur in the endoplasmatic reticulum, where classical class I molecules are loaded, but in autophagosomes (Camilli et al. 2016).

These considerations prompted a next set of experiments in which we tested proteolytic degradation of native and citrullinated MOG34-56 peptide in two preparations of human B cells, respectively, naïve B cells stimulated with CpG oligonucleotides that activate them via TLR9 or B lymphoblastoid cells infected with EBV (Morandi et al. 2017). Of note, data from Zou et al. (2012) revealed that infection of B cells with EBV reduces GatG expresssion to background level. Nevertheless, we observed that the native MOG34-56 peptide as well as the one citrullinated at the Arg^41^ position was rapidly degraded in the B cells. However, MOG34-56 peptide citrullinated at the Arg^46^ position was not (completely) degraded, especially when autophagy was boosted with rapamycin (Morandi et al. 2017). The presumed importance of the Arg^46^ position is that it lies within a putative F-LIR motif (^44^FSRV^47^) via which the peptide may bind to LC3, a docking molecule for specific sequestration of cargo in autophagomes (Birgisdottir et al. 2013). These observations led us to hypothesize that cleavage of the MOG34-56 peptide at the Arg^46^ residue may abrogate the autophagy escape route from cathepsin G exposure in phagolysomes, while conversion of this residue into citrulline abolishes cleavage and enables association of the peptide with autophagosomes ('t Hart et al. 2016).

## A Novel Pathogenic Role of EBV-Infected B Cell in the Marmoset EAE Model

A study in the mouse EAE model revealed that the Arg^41^ and Arg^46^ residues are essential contact points for binding of the epitope to the antigen receptor of encephalitogenic CD4^+^ T cells and that citrullination at these positions abrogates T cell recognition (Carrillo-Vico et al. 2010). The obvious paradox created by this observation, when translated to the MOG34-56/IFA model in marmosets, is that protection of the peptide against destructive processing in B cells by cathepsin G requires citrullination of at least the Arg^46^ residue, while this modification may abrogate T cell recognition. We asked therefore whether there might be another pathogenic mechanism at work in the model.

**Fig. 1:**
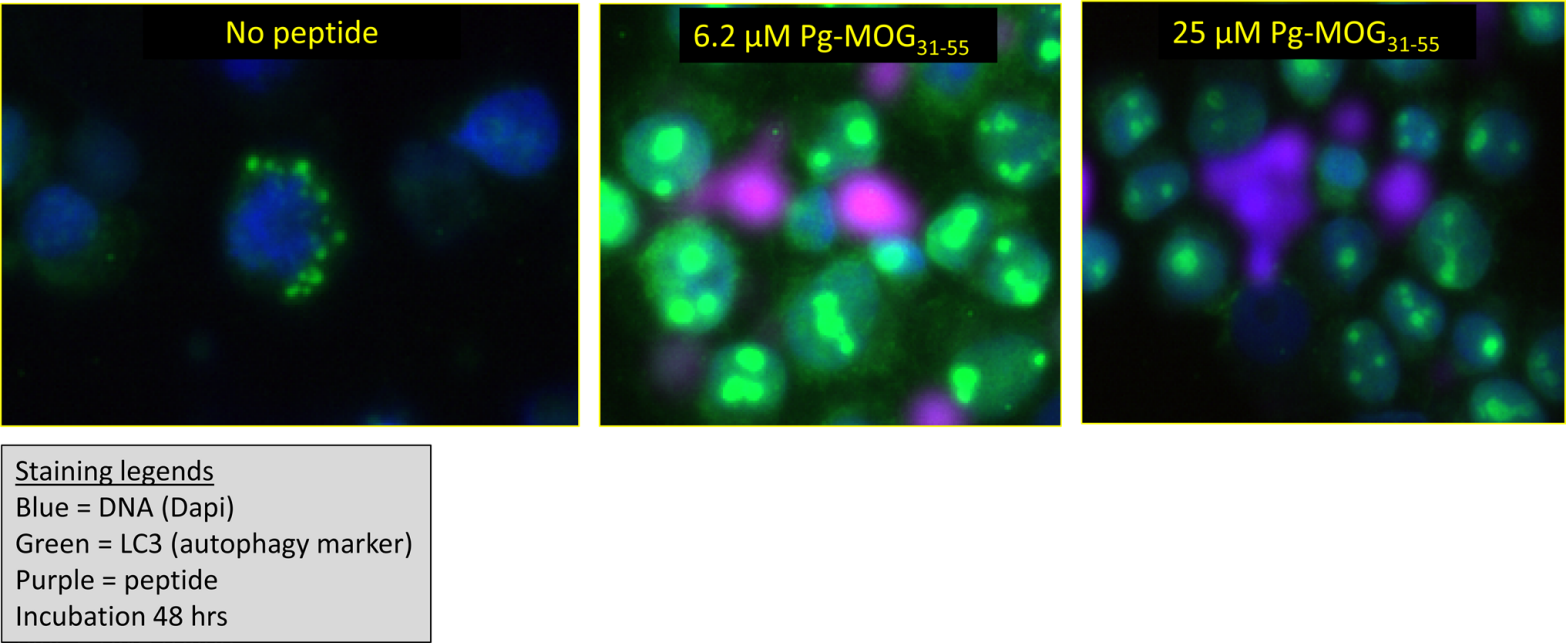
Feeding MOG31-55 peptide to EBV-infected human B cells boosts LC3 expression. Human EBV-infected B cells were incubated for 48 h with the indicated doses MOG31-55. The peptide was labeled with the bioorthogonal handle Pg to enable its visualization via click chemistry (Araman et al. 2019). After the incubation, the cells were washed and subsequently spun onto microscopy slides (28*g*). The slides were processed for immunostaining for LC3 as described (Araman et al. 2019). Colors: blue = cell nuclei; green = autophagosomes; purple = the MOG peptide

To test this possibility, we used a version of the peptide that could be visualized during and after the incubation with B cells via tagging a fluorochrome. This was achieved by coupling propargylglycine as a bioorthogonal handle to a slightly elongated variant of the MOG34-56 peptide, i.e., 31–55. Figure 1 shows a representative example of non-citrullinated MOG peptide incubated for 48 h with human EBV-infected B cells. Clearly, the peptide is not degraded, but seems to be extruded in an aggregated form. Concomittantly, LC3 expression is strongly increased. These features may be explained by a recently documented novel feature of citrullinated MOG peptide, namely the capacity to form toxic amyloid-like aggregates (Araman et al. 2019).

## Conclusion

Despite decades of intensive research, scientists have only superficial understanding of the critical processes in MS pathogenesis. Valuable insights can be gained from immunotherapy studies and by investigating the biological underpinning of MS risk factors, as these are very likely connected to rate-limiting steps in the disease process. The recognition that infection with EBV substantially enhances the risk of developing MS in genetically prone individuals (Ascherio and Munger 2015) converges with the observation in immunotherapy trials using anti-CD20 antibodies that the primary targets of the virus in the immune system, i.e. B cells, have a central pathogenic role in the disease (Barun and Bar-Or 2012).

Our research in the well-validated and translationally relevant marmoset EAE model revealed a central role of B cells in the control of autoimmunity against MOG, which seems to be exerted via destructive processing of the CD8 T cell epitope MOG40-48 by cathepsin G. This essentially tolerogenic mechanism is abolished when the B cells are infected with EBV. Analysis of the underlying mechanism revealed that the infection induces a post-translational modification in the epitope, i.e., replacement of Arg^46^ by citrulline, which enables association of the EAE inducing MOG34-56 peptide with autophagosomes. We hypothesized that via this route, the vulnerable MOG40-48 epitope may be protected against fast endolysosomal degradation by cathepsin G and that its loading onto Caja-E molecules may be promoted (‘t Hart et al. 2016).

Summarizing, we posit an important tolerogenic function of cathepsin G in the marmoset EAE model, namely to prevent the B cell-mediated activation of hazardous T cells by destroying their MOG40-48 autoantigen epitope during processing. Infection of the B lymphocytes with EBV induces cellular mechanisms, citrullination and autophagy, which protect the epitope against fast degradation, thereby enabling presentation to T cells. It would be highly interesting to test whether this principle can be extended to other autoantigens critical in other autoimmune diseases.

## References

[CR1] ‘t Hart BA, Bauer J, Muller HJ (1998). Histopathological characterization of magnetic resonance imaging- detectable brain white matter lesions in a primate model of multiple sclerosis: a correlative study in the experimental autoimmune encephalomyelitis model in common marmosets (*Callithrix jacchus*). Am J Pathol.

[CR2] ‘t Hart BA, Gran B, Weissert R (2011). EAE: imperfect but useful models of multiple sclerosis. Trends Mol Med.

[CR3] ‘t Hart BA, Jagessar SA, Haanstra K (2013). The primate EAE model points at EBV-infected B cells as a preferential therapy target in multiple sclerosis. Front Immunol.

[CR4] ‘t Hart BA, van Kooyk Y, Geurts JJ (2015). The primate autoimmune encephalomyelitis model; a bridge between mouse and man. Ann Clin Transl Neurol.

[CR5] ‘t Hart BA, Kap YS, Morandi E (2016). EBV infection and multiple sclerosis: lessons from a marmoset model. Trends Mol Med.

[CR6] ‘t Hart BA, Dunham J, Faber BW (2017). A B cell-driven autoimmune pathway leading to pathological hallmarks of progressive multiple sclerosis in the marmoset experimental autoimmune encephalomyelitis model. Front Immunol.

[CR7] Absinta M, Sati P, Reich DS (2016). Advanced MRI and staging of multiple sclerosis lesions. Nat Rev Neurol.

[CR8] Araman C, van Gent ME, Meeuwenoord NJ (2019). Amyloid-like behavior of site-specifically citrullinated myelin oligodendrocyte protein (MOG) peptide fragments inside EBV-iInfected B cells influences their cytotoxicity and autoimmunogenicity. Biochemistry.

[CR9] Ascherio A, Munger KL (2015). EBV and autoimmunity. Curr Top Microbiol Immunol.

[CR10] Barun B, Bar-Or A (2012). Treatment of multiple sclerosis with anti-CD20 antibodies. Clin Immunol.

[CR11] Ben-Nun A, Wekerle H, Cohen IR (1981). The rapid isolation of clonable antigen-specific T lymphocyte lines capable of mediating autoimmune encephalomyelitis. Eur J Immunol.

[CR12] Birgisdottir AB, Lamark T, Johansen T (2013). The LIR motif—crucial for selective autophagy. J Cell Sci.

[CR13] Brok HP, Uccelli A, Kerlero De Rosbo N (2000). Myelin/oligodendrocyte glycoprotein-induced autoimmune encephalomyelitis in common marmosets: the encephalitogenic T cell epitope pMOG24-36 is presented by a monomorphic MHC class II molecule. J Immunol.

[CR14] Burster T, Beck A, Tolosa E (2004). Cathepsin G, and not the asparagine-specific endoprotease, controls the processing of myelin basic protein in lysosomes from human B lymphocytes. J Immunol.

[CR15] Camilli G, Cassotta A, Battella S (2016). Regulation and trafficking of the HLA-E molecules during monocyte-macrophage differentiation. J Leukoc Biol.

[CR16] Carrillo-Vico A, Leech MD, Anderton SM (2010). Contribution of myelin autoantigen citrullination to T cell autoaggression in the central nervous system. J Immunol.

[CR17] Cho Y, Ramer J, Rivailler P (2001). An Epstein-Barr-related herpesvirus from marmoset lymphomas. Proc Natl Acad Sci USA.

[CR18] Fingeroth JD, Weis JJ, Tedder TF (1984). Epstein-Barr virus receptor of human B lymphocytes is the C3d receptor CR2. Proc Natl Acad Sci USA.

[CR19] Haanstra KG, Jagessar SA, Bauchet AL (2013). Induction of experimental autoimmune encephalomyelitis with recombinant human myelin oligodendrocyte glycoprotein in incomplete freund's adjuvant in three non-human primate species. J Neuroimmune Pharmacol.

[CR20] Hauser SL, Waubant E, Arnold DL (2008). B-cell depletion with rituximab in relapsing-remitting multiple sclerosis. N Engl J Med.

[CR21] Ireland JM, Unanue ER (2011). Autophagy in antigen-presenting cells results in presentation of citrullinated peptides to CD4 T cells. J Exp Med.

[CR22] Jagessar SA, Smith PA, Blezer E (2008). Autoimmunity against myelin oligodendrocyte glycoprotein is dispensable for the initiation although essential for the progression of chronic encephalomyelitis in common marmosets. J Neuropathol Exp Neurol.

[CR23] Jagessar SA, Kap YS, Heijmans N (2010). Induction of progressive demyelinating autoimmune encephalomyelitis in common marmoset monkeys using MOG34-56 peptide in incomplete freund adjuvant. J Neuropathol Exp Neurol.

[CR24] Jagessar SA, Heijmans N, Bauer J (2012). Antibodies against human BLyS and APRIL attenuate EAE development in marmoset monkeys. J Neuroimmune Pharmacol.

[CR25] Jagessar SA, Heijmans N, Blezer EL (2012). Unravelling the T-cell-mediated autoimmune attack on CNS myelin in a new primate EAE model induced with MOG34-56 peptide in incomplete adjuvant. Eur J Immunol.

[CR26] Jagessar SA, Fagrouch Z, Heijmans N (2013). The different clinical effects of anti-BLyS, anti-APRIL and anti-CD20 antibodies point at a critical pathogenic role of gamma-herpesvirus infected B cells in the marmoset EAE model. J Neuroimmune Pharmacol.

[CR27] Jagessar SA, Heijmans N, Blezer EL (2015). Immune profile of an atypical EAE model in marmoset monkeys immunized with recombinant human myelin oligodendrocyte glycoprotein in incomplete Freund's adjuvant. J Neuroinflammation.

[CR28] Jagessar SA, Holtman IR, Hofman S (2016). Lymphocryptovirus infection of nonhuman primate B cells converts destructive into productive processing of the pathogenic CD8 T cell epitope in myelin oligodendrocyte glycoprotein. J Immunol.

[CR29] Jiang W, Lederman MM, Harding CV (2011). Presentation of soluble antigens to CD8+ T cells by CpG oligodeoxynucleotide-primed human naive B cells. J Immunol.

[CR30] Kap YS, Smith P, Jagessar SA (2008). Fast progression of recombinant human myelin/oligodendrocyte glycoprotein (MOG)-induced experimental autoimmune encephalomyelitis in marmosets is associated with the activation of MOG34-56-specific cytotoxic T cells. J Immunol.

[CR31] Kap YS, van Driel N, Blezer E (2010). Late B cell depletion with a human anti-human CD20 IgG1kappa monoclonal antibody halts the development of experimental autoimmune encephalomyelitis in marmosets. J Immunol.

[CR32] Kap YS, Bauer J, Driel NV (2011). B cell depletion attenuates white and gray matter pathology in marmoset experimental autoimmune encephalomyelitis. J Neuropathol Exp Neurol.

[CR33] Kap YS, Jagessar SA, Dunham J (2016). The common marmoset as an indispensable animal model for immunotherapy development in multiple sclerosis. Drug Discov Today.

[CR34] Kappos L, Hartung HP, Freedman MS (2014). Atacicept in multiple sclerosis (ATAMS): a randomised, placebo-controlled, double-blind, phase 2 trial. Lancet Neurol.

[CR35] Khan G, Miyashita EM, Yang B (1996). Is EBV persistence in vivo a model for B cell homeostasis?. Immunity.

[CR36] Manoury B, Mazzeo D, Fugger L (2002). Destructive processing by asparagine endopeptidase limits presentation of a dominant T cell epitope in MBP. Nat Immunol.

[CR37] Meinl E, Hoch RM, Dornmair K (1997). Encephalitogenic potential of myelin basic protein-specific T cells isolated from normal rhesus macaques. Am J Pathol.

[CR38] Morandi E, Jagessar SA, t Hart BA (2017). EBV infection empowers human B cells for autoimmunity: role of autophagy and relevance to multiple sclerosis. J Immunol.

[CR39] Nossal GJ (1991). Molecular and cellular aspects of immunologic tolerance. Eur J Biochem.

[CR40] Raymond WW, Trivedi NN, Makarova A (2010). How immune peptidases change specificity: cathepsin G gained tryptic function but lost efficiency during primate evolution. J Immunol.

[CR41] Schluesener HJ, Wekerle H (1985). Autoaggressive T lymphocyte lines recognizing the encephalitogenic region of myelin basic protein: in vitro selection from unprimed rat T lymphocyte populations. J Immunol.

[CR42] Serre L, Girard M, Ramadan A (2017). Thymic-specific serine protease limits central tolerance and exacerbates experimental autoimmune encephalomyelitis. J Immunol.

[CR43] Smith PA, Heijmans N, Ouwerling B (2005). Native myelin oligodendrocyte glycoprotein promotes severe chronic neurological disease and demyelination in Biozzi ABH mice. Eur J Immunol.

[CR44] Villoslada P, Abel K, Heald N (2001). Frequency, heterogeneity and encephalitogenicity of T cells specific for myelin oligodendrocyte glycoprotein in naive outbred primates. Eur J Immunol.

[CR45] von Budingen HC, Palanichamy A, Lehmann-Horn K (2015). Update on the autoimmune pathology of multiple sclerosis: B cells as disease-drivers and therapeutic targets. Eur Neurol.

[CR46] Zou F, Schmon M, Sienczyk M (2012). Application of a novel highly sensitive activity-based probe for detection of cathepsin G. Anal Biochem.

